# A model-based approach to selection of tag SNPs

**DOI:** 10.1186/1471-2105-7-303

**Published:** 2006-06-15

**Authors:** Pierre Nicolas, Fengzhu Sun, Lei M Li

**Affiliations:** 1 Molecular and Computational Biology Program, Department of Biological Sciences, University of Southern California, Los Angeles, USA; 2 Mathématique, Informatique et Génome, INRA, Jouy-en-Josas, France; 3 Department of Mathematics, University of Southern California, Los Angeles, USA

## Abstract

**Background:**

Single Nucleotide Polymorphisms (SNPs) are the most common type of polymorphisms found in the human genome. Effective genetic association studies require the identification of sets of tag SNPs that capture as much haplotype information as possible. Tag SNP selection is analogous to the problem of data compression in information theory. According to Shannon's framework, the optimal tag set maximizes the entropy of the tag SNPs subject to constraints on the number of SNPs. This approach requires an appropriate probabilistic model. Compared to simple measures of Linkage Disequilibrium (LD), a good model of haplotype sequences can more accurately account for LD structure. It also provides a machinery for the prediction of tagged SNPs and thereby to assess the performances of tag sets through their ability to predict larger SNP sets.

**Results:**

Here, we compute the description code-lengths of SNP data for an array of models and we develop tag SNP selection methods based on these models and the strategy of entropy maximization. Using data sets from the HapMap and ENCODE projects, we show that the hidden Markov model introduced by Li and Stephens outperforms the other models in several aspects: description code-length of SNP data, information content of tag sets, and prediction of tagged SNPs. This is the first use of this model in the context of tag SNP selection.

**Conclusion:**

Our study provides strong evidence that the tag sets selected by our best method, based on Li and Stephens model, outperform those chosen by several existing methods. The results also suggest that information content evaluated with a good model is more sensitive for assessing the quality of a tagging set than the correct prediction rate of tagged SNPs. Besides, we show that haplotype phase uncertainty has an almost negligible impact on the ability of good tag sets to predict tagged SNPs. This justifies the selection of tag SNPs on the basis of haplotype informativeness, although genotyping studies do not directly assess haplotypes. A software that implements our approach is available.

## Background

Genetic association studies at the population level are one of the most promising ways to discover the genetic basis of subtle human phenotypes such as complex diseases or drug responses [1-3]. The aim of these studies is to map genetic factors underlying such phenotypes by comparing genetic information and phenotypes of individuals sampled from a population. As whole-genome sequencing for each individual remains currently impossible, the genetic information is typically assessed through a set of genetic markers that carry information on their neighborhoods due to Linkage Disequilibrium (LD). Single Nucleotide Polymorphisms (SNPs), the most common type of polymorphisms in the human genome, are markers of great interest in this context. In fact, they are so common that the information they carry seems highly redundant in high LD regions of the human genome. Consequently, it makes sense to select a small fraction of the SNPs, the tag SNPs, for mapping purposes. This can significantly reduce genotyping effort without much loss of power [4]. One of the main goals of the international HapMap project is to acquire the knowledge of the LD structure needed for the choice of efficient tag SNPs [5].

The stake in the choice of the tag SNPs has driven the development of numerous methods for their selection during the past few years. These methods were reviewed by Halldórsson *et al*. [6]. In their view, methods differ mostly in two major aspects: the quality or correlation measure used for the definition of tag SNPs and the algorithm used for the minimization of the final number of tag SNPs. For instance, we can seek a subset of SNPs such that every SNP that does not belong to the tag set (*i.e. *a tagged SNP) has a *r*^2 ^measure of pairwise LD with a tag SNP greater than a given threshold [7,8]. Some studies suggest that the human genome is composed of haplotype blocks with high LD and relatively limited haplotype diversity, separated by short regions of low LD [9-11]. The concept of block has immediately received a great deal of attention in the context of tag SNP selection because a block may contain a large number of SNPs, but a few SNPs are enough to uniquely identify the haplotypes in a block. A straightforward block-based strategy consists of two separate steps: (1) identify haplotype blocks and (2) select the tag SNPs [9]. In one of the most popular tag SNP selection approaches introduced by Zhang *et al*. [12], both the selection of block boundaries and the choice of tag SNPs are optimized jointly to capture the majority of haplotype diversity within blocks. The idea is implemented in the HapBlock software [13]. Here, rather than a "hard" definition of blocks, we describe LD by "soft" parameter values in an appropriate probabilistic model. Our choice of a block-free perspective is motivated by numerous observations on block partitioning documented in the literature. The most important reason is that although some regions of the human genome seems to conform quite well to a description in terms of blocks, other regions do not [14]. It also appears that the definition of a block is not straightforward and very different partitioning are obtained depending on the adopted definition, leading to the selection of very different number of tag SNPs [15]. In addition, block partitioning has been reported to be affected by many factors such as the SNP density, the number of observed sequences and the choice of a particular set of markers [16-18]. A good probabilistic model of the haplotype sequences can better capture LD patterns than haplotype blocks or simple measures of pairwise LD do. We also find two other benefits in adopting a model-based point of view. First, it allows us to tackle the tag SNP selection problem as a data compression problem using the widely accepted Shannon's measure of information content. Second, a probabilistic model provides the machinery to predict tagged SNPs from tag ones. Effectiveness of tag sets can then be evaluated through their information content and their ability to predict other SNPs, both measurements requiring a good probabilistic model. This direct measure of performance makes it possible to compare various methods, including model-based and other tagging methods. Similar ideas can be found in some previous studies. In particular, the formulation of the haplotype tag SNP selection as a data compression problem and the idea of evaluating tag sets through their prediction performances are advocated in Halldórsson *et al*. [19] and Shannon entropy based criteria to measure LD and to select tag SNPs are described in [18] and [20], respectively. However, overall, little attention has been paid to selection and use of good probabilistic models for tag SNP selection.

The coalescent model [21] and its generalizations are the most appealing approaches to relating genetic variation to recombination rate and other genetic and demographic factors. However, the inference based on coalescence is computationally challenging. Rather, we consider approximations that are computationally tractable. We start with a simple Markov model that has fast algorithms for exact solutions and can serve as a baseline reference. Next we consider several hidden Markov models (HMMs). In particular, we study the model introduced by Li and Stephens in the context of recombination rate estimation [22]. We show how entropy under different models of haplotype sequences can be maximized in practice. As another reference, we propose a greedy method that maximizes entropy without the linear structure embedded in Markov models and HMMs.

Model-based SNP tagging hinges on the issue of model comparison. Criterion such as AIC [23] and BIC [24] have been proposed in the literature for model selection. In this article, to deal with various complicated models, we adopt the principle of Minimum Description Length (MDL), which unifies the likelihood theory and Shannon's theory [25]. According to the MDL principle, the best model gives the shortest description code-length of the data. Analytical forms of description code-lengths are only available for relatively simple models [26]. The two-stage coding scheme has been adopted to delineate haplotype blocks using MDL after formulating the task as the choice of a model whose parameter space size increases smoothly with the number of blocks [27-29]. Here, we compare a relatively small number of models with very different parameter spaces and thus we prefer to evaluate the description code-length of the models by using a cross-validation version of the predictive coding scheme for bit-counting that automatically penalizes over-fitting.

We compare the performances of the models and of the resulting tag sets on haplotype data from the international HapMap and ENCODE projects. The performances of the tag sets selected by our model-based method are also compared with tag sets selected by the HapBlock software [13], with tag sets selected by the method proposed by Carlson *et al*. [8], with SNPs chosen at random, and with evenly spaced SNPs. Finally, we assess the loss of predictive power of the tag sets due to the typing of genotypes instead of haplotypes.

## Results

### Model-based tag SNP selection

Let *X *= {*X*_*j*_, *j *= 1, 2, ... , *n*} denote the random variable that corresponds to a haplotype sequence. When selecting a subset of tag SNPs indexed by *J *⊂ {1, 2,..., *n*}, we want to minimize the loss of information when we experimentally assess the subset *J *instead of the whole set of SNPs. In other words, we would like to find the optimal compression *X*_*J *_of *X*. Shannon has shown that the information content, or randomness, of *X *is well measured by its entropy defined as *H*(*X*) = -E
 MathType@MTEF@5@5@+=feaafiart1ev1aaatCvAUfKttLearuWrP9MDH5MBPbIqV92AaeXatLxBI9gBaebbnrfifHhDYfgasaacH8akY=wiFfYdH8Gipec8Eeeu0xXdbba9frFj0=OqFfea0dXdd9vqai=hGuQ8kuc9pgc9s8qqaq=dirpe0xb9q8qiLsFr0=vr0=vr0dc8meaabaqaciaacaGaaeqabaqabeGadaaakeaatuuDJXwAK1uy0HMmaeHbfv3ySLgzG0uy0HgiuD3BaGabaiab=ri8fbaa@388C@_*X *_log ℙ(*X*) [30]. For any given subset *J*, the information content of *X *can be decomposed into two parts using the chain rule of entropy: *H*(*X*) = *H*(*X*_*J*_) + *H*(XJ˜
 MathType@MTEF@5@5@+=feaafiart1ev1aaatCvAUfKttLearuWrP9MDH5MBPbIqV92AaeXatLxBI9gBaebbnrfifHhDYfgasaacH8akY=wiFfYdH8Gipec8Eeeu0xXdbba9frFj0=OqFfea0dXdd9vqai=hGuQ8kuc9pgc9s8qqaq=dirpe0xb9q8qiLsFr0=vr0=vr0dc8meaabaqaciaacaGaaeqabaqabeGadaaakeaacqWGybawdaWgaaWcbaGafmOsaOKbaGaaaeqaaaaa@2F3D@|*X*_*J*_), where the entropy *H*(*X*_*J*_) is the information carried by the subset *J *and the conditional entropy *H*(XJ˜
 MathType@MTEF@5@5@+=feaafiart1ev1aaatCvAUfKttLearuWrP9MDH5MBPbIqV92AaeXatLxBI9gBaebbnrfifHhDYfgasaacH8akY=wiFfYdH8Gipec8Eeeu0xXdbba9frFj0=OqFfea0dXdd9vqai=hGuQ8kuc9pgc9s8qqaq=dirpe0xb9q8qiLsFr0=vr0=vr0dc8meaabaqaciaacaGaaeqabaqabeGadaaakeaacqWGybawdaWgaaWcbaGafmOsaOKbaGaaaeqaaaaa@2F3D@|*X*_*J*_) = -E
 MathType@MTEF@5@5@+=feaafiart1ev1aaatCvAUfKttLearuWrP9MDH5MBPbIqV92AaeXatLxBI9gBaebbnrfifHhDYfgasaacH8akY=wiFfYdH8Gipec8Eeeu0xXdbba9frFj0=OqFfea0dXdd9vqai=hGuQ8kuc9pgc9s8qqaq=dirpe0xb9q8qiLsFr0=vr0=vr0dc8meaabaqaciaacaGaaeqabaqabeGadaaakeaatuuDJXwAK1uy0HMmaeHbfv3ySLgzG0uy0HgiuD3BaGabaiab=ri8fbaa@388C@_*X *_log ℙ(XJ˜
 MathType@MTEF@5@5@+=feaafiart1ev1aaatCvAUfKttLearuWrP9MDH5MBPbIqV92AaeXatLxBI9gBaebbnrfifHhDYfgasaacH8akY=wiFfYdH8Gipec8Eeeu0xXdbba9frFj0=OqFfea0dXdd9vqai=hGuQ8kuc9pgc9s8qqaq=dirpe0xb9q8qiLsFr0=vr0=vr0dc8meaabaqaciaacaGaaeqabaqabeGadaaakeaacqWGybawdaWgaaWcbaGafmOsaOKbaGaaaeqaaaaa@2F3D@|*X*_*J*_) is the information loss due to the residual randomness of the SNPs J˜
 MathType@MTEF@5@5@+=feaafiart1ev1aaatCvAUfKttLearuWrP9MDH5MBPbIqV92AaeXatLxBI9gBaebbnrfifHhDYfgasaacH8akY=wiFfYdH8Gipec8Eeeu0xXdbba9frFj0=OqFfea0dXdd9vqai=hGuQ8kuc9pgc9s8qqaq=dirpe0xb9q8qiLsFr0=vr0=vr0dc8meaabaqaciaacaGaaeqabaqabeGadaaakeaacuWGkbGsgaacaaaa@2DD8@ that do not belong to *J*. In this framework, we seek a set of tag SNPs *J *that maximizes *H*(*X*_*J*_) subject to the constraint on the number of markers in *J*. The same subset *J *minimizes *H*(XJ˜
 MathType@MTEF@5@5@+=feaafiart1ev1aaatCvAUfKttLearuWrP9MDH5MBPbIqV92AaeXatLxBI9gBaebbnrfifHhDYfgasaacH8akY=wiFfYdH8Gipec8Eeeu0xXdbba9frFj0=OqFfea0dXdd9vqai=hGuQ8kuc9pgc9s8qqaq=dirpe0xb9q8qiLsFr0=vr0=vr0dc8meaabaqaciaacaGaaeqabaqabeGadaaakeaacqWGybawdaWgaaWcbaGafmOsaOKbaGaaaeqaaaaa@2F3D@|*X*_*J*_) and maximizes the mutual information between *X*_*J *_and *X*.

The method of entropy maximization requires the specification of a probabilistic model for *X*. Better models provide more accurate quantification of the information content contained in sets of SNPs and thus are also expected to allow the selection of better tag SNPs. In the Methods section we describe the models along with tractable tag SNP selection procedures for each of them.

### Model comparison using description code-lengths

Each model provides a coding system that can be used to encode haplotype sequences. When averaged over many new sequences, the length of this code gives an assessment of the quality of the model: the shorter the code-length, the better the model (MDL principle). Code-lengths are closely related to the information content of the data (see [31] for a didactic presentation). Both quantities are usually expressed in bits and any achievable code-length is an upper bound to the real information content of the data. Code-length equals to the information content only in the idealistic case where the "true" model is used for encoding. In Table 1, we show the cross-validated estimates of the code-lengths computed as the negative cross-log-likelihoods (logarithm to base 2), for the ten ENCODE data sets and the chromosome 7 data set (data sets are described in the Methods section). Code-lengths are expressed in bits and thus are directly comparable to the number of SNPs in each data set. For instance, the Li and Stephens model that accounts for variable recombination rates gives a 52-bit long description per haplotype of the 1134 SNPs in the ENr112 region from the CEU population: the same description length as that of the outcome of 52 fair coin tosses.

The simple unconstrained two-state HMM shortens the code-lengths by about 30% compared to the simple Markov model. The model introduced by Daly *et al*. (abbreviated in the sequel as HMM-4D) is a better choice. It is better than the "greedy" model with context-size 1 (GR-1) on chromosome-scale data sets but worst on most ENCODE regions suggesting that the relative performances of the two models depend on SNP density and/or minor alleles frequencies of the SNPs. The "greedy" model with context-size 2 (GR-2) systematically outperforms these two models.

In all cases, Li and Stephens models with homogeneous recombination rate (LS-HOM) and with heterogeneous recombination rate (LS-HET) stand out by their short description lengths. The shortest description length is always achieved by LS-HET that shortens the code-lengths by about 40% compared to the GR-2 model. The amplitudes of the differences between code-lengths associated to LS-HET and LS-HOM vary greatly depending on the genomic region. It is less than 2% in the ENm013 and ENrl23 regions and more than 8% in the ENrl3l and ENr232 regions, being around 5% for the chromosome-scale data set (Chromosome 7). These differences may reflect variations in the pattern of recombination. In addition to demonstrating a strong superiority of the Li and Stephens HMMs, code-lengths also reveals some interesting features of the data. We note in particular that the information content, or randomness, as assessed with the best model (LS-HET) is systematically higher for YRI haplotypes than for CEU haplotypes. For instance, the difference is 44% on the Chromosome 7 data set. We also observe that the information content measured in bits is always much smaller than the number of SNPs: a prerequisite for the existence of small tag sets capturing most of the information.

### Informativeness versus number of tag SNPs

**Figure 1:**
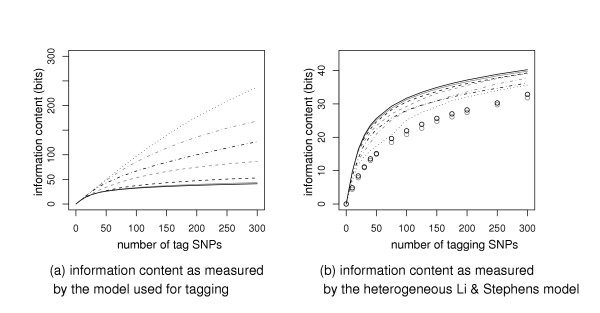
Information content versus the number of tag SNPs in the ENr112 region from CEU population. (a) The information content is computed according to the model used to select the tag SNPs. Top-down: Markov model (dotted black line), unconstrained and Daly *et al*. HMMs (dot-dashed gray and black lines, respectively), models with context sizes 1 and 2 (dashed gray and black lines, resp.), homogeneous and heterogeneous Li and Stephens HMMs (solid gray and black lines, resp.). (b) The information content is always computed according the heterogeneous Li and Stephens model, regardless of the tag SNP selection method. The symbols for the tag SNP selection methods are the same as in (a). Additional symbols show the information content of evenly spaced tag SNPs (black circles), and randomly chosen tag SNPs (gray circles).

Next we check how informativeness increases as we select more tag SNPs. We emphasize that the calculation of informativeness does not necessarily depend on the tagging method and should in fact be measured according to the best available model (*i.e. *LS-HET) when comparing tag sets. Figure 1 shows the information content versus number of tag SNPs in the ENr112 region from the CEU population. We do not present the results for other regions as they look qualitatively similar.

In Figure 1 (a), the information content is computed by the same model as used to select the tag SNPs. As expected the information content increases as more SNPs are selected. Let us look at the two extreme cases. In the Li and Stephens models, the information curve increases at a much slower pace after 75 SNPs are selected. On the other end, in the simple Markov model, the information content keeps growing rapidly as more SNPs are selected. With the results of model comparison in mind, we know that Li and Stephens models better describe the true information pattern. This supports the hypothesis of high redundancy in SNP information. Those extra bits seen in the simple Markov model reflect its relatively low efficiency in encoding haplotypes.

In Figure 1 (b), the information is computed with the LS-HET model. As expected, tag sets selected with LS-HET are the most informative. The informativeness of a tag set can be thought as a measure of its ability to describe SNP data. If we compare models according to this measure, the results are consistent with those obtained using cross-validated code-lengths although differences between tag sets are smaller than differences between models. Averaging information content over all ENCODE regions from both CEU and YRI, we find that 100 SNPs selected with LS-HET capture the same amount of information as 108 SNPs selected with LS-HOM or 113 SNPs selected with GR-2, next come GR-1 (120 SNPs) and HMM-4D (150 SNPs).

We also study tag sets made of evenly spaced SNPs and randomly picked SNPs (Figure 1 (b)). They appear to be less informative. The poor performance of these tag SNPs is particularly evident for small tag sets. The information content of the 20-SNPs tag set selected by the heterogeneous Li and Stephens model is about 16 bits whereas it is only about 8 bits for random or evenly spaced SNPs. When a model is used to select tag SNPs, the information increases at the rate of about one bit per SNP at the very beginning because any model starts by choosing approximately independent SNPs with high minor allele frequencies. The rates slow down as more SNPs are selected: the SNPs that do not belong to the tag set tend to have strong dependence with tag SNPs or lower minor alleles frequencies.

### Prediction of tagged SNPs

**Figure 2:**
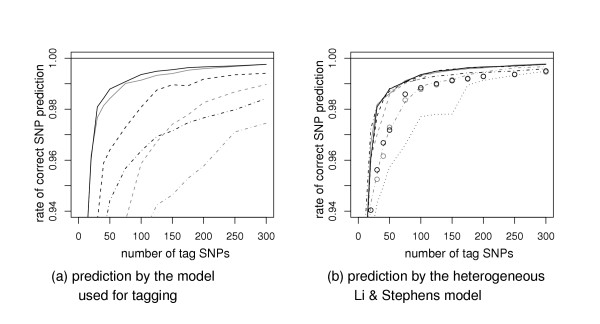
Rates of correct SNP prediction versus the number of tag SNPs in the ENr112 region from CEU population. (a) The prediction is performed with the model used to select the tag SNPs. Tag SNP selection methods are indicated with the same symbols as in Figure 1 (a). The performance of the Markov model is not shown, (b) The predictions is performed by the LS-HET model, regardless of the tag SNP selection method. The symbols are the same as in Figure 1 (b).

A good tag SNP set should allow us to predict the allelic status of the tagged SNPs with high accuracy. Explicitly or implicitly, a prediction procedure is usually associated with a probabilistic model. Here, we take the most probable allele, given a model and the haplotype known at the tag SNPs, as our prediction. These probabilities are easy to compute in the HMM framework. Predictions based on greedy models rely on the observed frequencies in 1000 simulations of the tagged SNPs given the haplotype at the tag SNPs. Figure 2 shows the fraction of correctly predicted SNPs in the ENr112 region from the CEU population. In Figure 2 (a), predictions are based on the same model as used to select the tag SNPs. The relative standings of these curves are similar to those in Figure 1, although their curvatures are different. Once again, the Li and Stephens models stand out. The correct prediction rates are as high as 99.0% and 99.1% with 75 tag SNPs when LS-HOM and LS-HET are used for both SNP tag and SNP prediction, respectively. This rate is 97.3% for the GR-2 model and next it drops to 95.7% and 93.6% for the HMM-4D and GR-1 models.

Figure 2 (b) presents results that help distinguish between the contribution of the tag set and the contribution of the prediction method to the rate of correct prediction: we take LS-HET as our prediction method no matter how the tag set was selected. Thereby the prediction accuracy gives a direct measure of the predictive value of a tag set. Overall, differences among tag sets are much smaller than those reported in Figure 2 (a). With 75 tag SNPs, the rate of correct prediction is 99.1% for the tag sets selected by LS-HOM, GR-2 or HMM-4D. GR-1 tag set comes next with a 99.0% prediction accuracy.

The comparisons between tag sets on the other data sets show similar patterns for the relative standings of the rate of correct prediction. However, it is worth mentioning that rates correct prediction achieved on CEU data sets are higher than those obtained on YRI data sets with the same number of tag SNPs. For instance, on average over all ENCODE regions, 75 LS-HET tag SNPs allow a prediction accuracy of 99.0% on CEU ENCODE regions but only 98.2% on YRI ENCODE regions where about 140 SNPs are needed to achieved an average of 99.0% of correct predictions. As expected, the fraction of SNPs that need to be retained in the tag set to obtain a desired level of correct prediction depend also strongly on the density of SNPs. On the chromosome 7 data set, where the average spacing between SNPs is about 6 times higher than in ENCODE regions, about 11000 SNPs are needed to obtain a 99.0% correct prediction rate on the CEU data set (17000 SNPs on the YRI data set).

### Comparison with other methods

**Figure 3:**
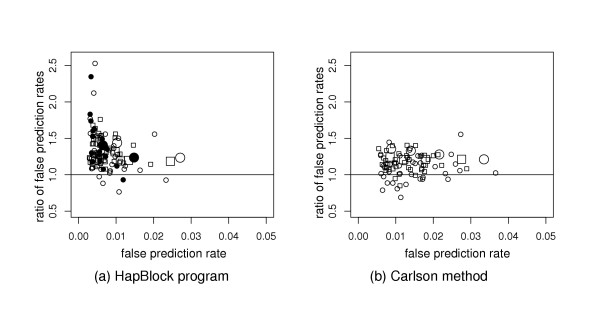
Comparison with other tag SNP selection algorithms. Performances of tag sets selected with (a) the HapBlock program, or (b) the Carlson method are compared with the performances tag sets of the same sizes selected with the LS-HET model. The horizontal axis reports the false prediction rate associated with the tag sets selected by the HapBlock program or the Carlson method. The vertical axis reports the ratio of this false prediction rate over the false prediction rate associated with the tag set of the same size selected with the LS-HET model. All the predictions are performed by the LS-HET model and the false prediction rates are averaged over the six cross-validation experiments. Each point corresponds to the combination of one data set and one set of user-defined parameters. Large symbols emphasize results obtained on Chromosome 7; circles and rectangles stand for results on CEU and YRI data sets, respectively. (a) For the HapBlock software, (*α*_*HB*_, *β*_*HB*_) are: (0.8, 0.099), (0.9, 0.099), (0.9, 0.049) and (0.95, 0.049) with the diversity criterion for tag SNP selection (open symbols); and (0.8, 0.099) and (0.9, 0.099) with the entropy criterion for tag SNP selection (close symbols). (b) The *r*^2 ^thresholds for the Carlson method are: 0.35, 0.5, 0.65 and 0.80.

We compare our best tag SNP selection method based on the LS-HET model with two methods previously described in the literature: the block-based dynamic algorithm implemented in the HapBlock software [13] and the Carlson *et al*. method [8]. Figure 3 presents results obtained on the different regions with different sets of user-defined parameters.

Figure 3 (a) reports the comparison with the HapBlock software in terms of rate of false prediction. We see that HapBlock tag sets are better than our tag sets in only 5 cases out of 111 (4.5%, p-value ≈ le – 25 by the non-parametric sign-test). On average, the ratio between the false prediction rate of the HapBlock tag sets and that of our tag sets is 1.36 for the ten ENCODE regions and 1.27 for Chromosome 7.

Figure 3 (b) reports the comparison with Carlson *et al*. method. The tag sets selected by the Carlson *et al*. method are better than our tag sets in only 14 cases out of 88 (16%, p-value ≈ le – 10 by the non-parametric sign-test). The average ratios between the false prediction rate of the Carlson *et al*. tag sets and that of our tag sets is 1.13 for the 10 ENCODE regions and 1.26 for Chromosome 7.

In addition to the better predictive power, the information content as measured by LS-HET was always higher for our tag sets than for the tag sets selected using either the HapBlock or the Carlson *et al*. method (data not shown).

### Genotyping versus haplotyping

The methods described above assume known haplotype information. However, most current typing platforms directly measure genotypes but not haplotypes. This means that the genotype at each typed SNP is experimentally known but not the phasing between alleles at different loci. Optimizing the tag set in the genotyping context may have to take into account the possibility that some SNPs are more informative than others for inferring haplotypes at the tag SNP loci. Such an optimization is not in the scope of this paper and would be computationally challenging if we do not want to sacrifice the necessary sophistication of a good haplotype model. Instead, we propose to assess the loss of predictive power due to phase uncertainty at the tag SNP loci by comparing the ability of our tag set to predict tagged SNP genotypes from either haplotypes or genotypes at the tag SNP loci.

The task of genotype modelling does not fundamentally differ from haplotype modelling as a genotype is simply a pair of haplotype whose phase is unknown (assuming Hardy-Weinberg equilibrium). Therefore, the best genotype model is just a modified version of the best haplotype model. Naturally we use the LS-HET model in this context: the LS-HET model generalizes rather easily to genotype sequences (see [32]); prediction is performed after computing the posterior probability of each genotype at each SNP with the forward-backward algorithm for HMMs.

**Figure 4:**
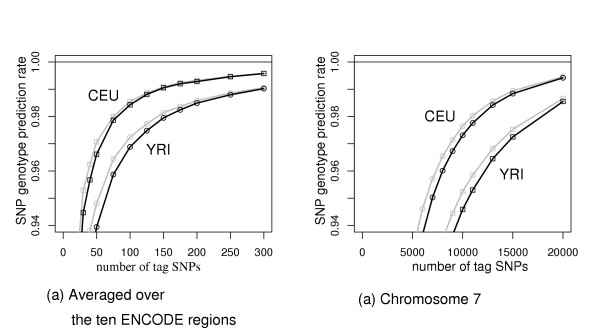
Assessment of the loss in prediction accuracy due to tag set phase uncertainty using the LS-HET model. Black lines stand for genotype predictions from tag set genotypes and gray lines for predictions from tag set haplotypes. Both curves are shown for the CEU and YRI populations.

Figure 4 shows the ability of our LS-HET tag sets to predict the genotypes when haplotypes at the tag SNPs loci are known (phase known) and when only genotypes are known (phase unknown). It appears that little predictive power is lost due to phase uncertainty as soon as the tag sets are reasonably large. For instance, on ENCODE regions from the CEU population, the genotype is predicted correctly at 98.0% of the positions with 75 SNPs when haplotypes are given and 97.9% when only genotypes are known (Figure 4 (a)). On chromosome 7 from CEU population, the rate of correct prediction decreases from 98.0% to 97.8% at 11000 tag SNPs (Figure 4 (b)).

## Discussion

It has been proposed to use Shannon entropy to assess LD [18] and to select tag SNPs [20] without relying on explicit models of haplotype sequences. The model-free approach relies on direct estimation of the entropy from the empirical haplotype frequencies and is not scalable to large set of SNPs. When SNP sets are large enough any haplotype is observed at most only once which causes the empirical estimate of Shannon entropy to plateau to log *Q*, with *Q *being the number of sequences sampled. In comparison, explicit modelling of the haplotypes makes it possible to estimate Shannon entropy whatever the size of the SNP set. In this context, appropriate models can bypass the concept of blocks and still account for simultaneous correlations between multiple markers.

The Li and Stephens models are becoming widely used in contexts including recombination rate estimation [22,33,34] and haplotype phase reconstruction [32]. This study is the first that reports their use in the context of tag SNP selection and it confirms their strengths. They outperform the other models in several aspects: description code-length of data, informativeness of tag SNPs, and prediction of tagged SNPs. Furthermore, we show that the heterogeneous version of the model, accounting for fine scale variations in the recombination rate, is actually better than the homogeneous version. This was not a *priori *obvious because the heterogeneous model has many more parameters than the homogeneous model and their inference could be unreliable. It should be noted that these parameters are closely related to the recombination rates [22] which are known to be hard to estimate [35]. However, we do not see a large difference between the heterogeneous and the homogeneous Li and Stephens model for the practical purpose of tag SNP selection. This may suggest that inference of the fine scale variations in the recombination rate, and in particular of the location and the intensity of the recombination hot-spots [33,36], is not very important in the context of tag SNP selection. The relatively good performances of our models of context-size 1 or 2 might encourage more work on related models such as graphical models for SNP data [37].

In this study we consider the problem of haplotype tag SNP selection assuming that haplotypes are available. In reality, the data are usually just genotype. Genotype trios give much more information on the haplotypes but they necessitate more genotyping and are impractical in most large case-control studies because of difficulties to find suitable trios. As in most of the works on haplotype tag SNP selection, the problem can be circumvented by using haplotypes inferred from genotype data. Examples of methods for inferring haplotypes from genotype data, or phase reconstruction, include those proposed by Excoffier and Slatkin [38], Niu *et al*. [39] and Stephens and Scheet [32]. However, these methods are not error-free and this may cause a problem at two levels. First, the choice of the tag SNPs relies on a set of haplotypes and could be sensitive to phase errors in these haplotypes. We do not believe this to be a very important concern as (1) a high density of SNP will typically be assessed and thus will permit accurate haplotype reconstruction and (2) trio genotyping is possible if haplotype reconstruction actually appear to be a problem. Second, the set of tag SNPs may not be optimal for phase reconstruction. Our results rule out this concern by showing that the impact of phase uncertainty on the ability to predict tagged SNPs from a good tag set is marginal. Even a very hypothetical tag set that could simultaneously remove any phase uncertainty and preserve the maximal amount of information on the tagged SNPs would not perform more than a few percent better than our tag sets. In addition, one may argue that if the tag set were genotyped in many individuals, these genotypes may be used to further reduce the phase uncertainty at the tag SNP loci.

Our results show that tagged SNPs can be predicted with a very high accuracy using a good tag set and a good prediction method. For instance, 1000 SNPs mapping a 500 Kbp long ENCODE regions from CEU population can be predicted with an average error rate of 1% using 75 tag SNPs. Rough computations suggest that if the 3 Gbp of whole genome was mapped at the same SNP density (6,000,000 SNPs), 500,000 tag SNPs would be enough to predict the other SNPs with a 1% average error rate. Due to the more complex haplotype structure of African populations [11,40], achieving the same rate for YRI population could require about twice as many tag SNPs. It would be interesting to investigate whether or not one should use these predictions in association studies. The answer may not be evident. On one hand, working with a smaller set of SNPs reduces the dimension of the analysis and thus it could be advantageous to restrict the analysis to the tag SNPs. On the other hand, tag SNP selection relies on knowledge about the haplotype structure that is latter lost if predictions are not used. Moreover, when the association study rely on single marker analyses where each SNP is tested separately, then it is probably worth to first predict tagged SNP to increase the probability of finding a single marker heavily linked to the causal SNP. Throughout this study we select tag sets by maximizing entropy given a model. The use of this quantity is motivated by strong arguments from information theory. If we compare how entropy and global prediction accuracy measure the power of tag sets, results suggest that entropy is a more sensitive quantity than prediction accuracy. For instance, the comparison between the average rate of correct prediction achieved with a tag set selected using the best model (LS-HET) and relatively simple models (GR-2, and even GR-1 or HMM-4D) show quite similar performances for each tag sets whereas the superiority of the Li and Stephens model is clearly established both in terms of code-lengths and predictive ability. We also see that the pace at which entropy increases with the number of tag SNPs does not slow down as quickly as the rate of correct prediction. We note that both measures are adopted in the popular machine learning tool of classification and regression tree (CART) [41]. Namely, entropy is used to generate more partition nodes while prediction (classification) accuracy is used to prune decision trees. An important issue in association studies is the choice of the number of tag SNPs. It may be a good idea to adopt the strategy used in the CART methodology for tag SNP selection. That is, after having pre-selected a tag set (possibly larger than needed) on the basis of entropy, we could determine the final number of tag SNPs by shrinking the original set on the basis of the prediction accuracy. In this context, it is worth mentioning that tag SNPs selected from a given collection may better predict SNPs from this collection than other SNPs [18]. Therefore, it could be sound in future works to set aside a fraction of the SNPs during the tag SNP selection process that could latter be used in order to choose the final number of tag SNPs on the basis of the prediction accuracy.

Finally, we were able to provide evidence of the advantage of our best tag SNP selection method over both the methods implemented in the HapBlock software and the method proposed by Carlson *et al*. [8]. We did that by comparing the performances of tag sets on their abilities to predict the tagged SNPs (as well as on the basis of their information content). These comparisons are meaningful because they rely on a good probabilistic model. In the future, this scheme that allows direct comparison of the intrinsic performances of tagging sets should be useful to further clarify the issues associated to the choice of tag SNPs.

## Conclusion

Our study provides strong evidence that the tag sets selected by our best method, based on Li and Stephens model, outperform those chosen by several existing methods. The results also suggest that information content evaluated with a good model is more sensitive for assessing the quality of a tagging set than the correct prediction rate of tagged SNPs. Besides, we show that haplotype phase uncertainty has an almost negligible impact on the ability of good tag sets to predict tagged SNPs. This justifies the selection of tag SNPs on the basis of haplotype informativeness, although genotyping studies do not directly assess haplotypes.

## Availability and requirements

A program that implements tag SNP selection, entropy computation, and tagged SNP prediction based on Li and Stephens HMMs is freely available under the terms of the GNU Public Licence at  and is also attached to this publication [see Additional file 1]. The programs implementing the other tag SNP selection methods described in this paper will be made available upon request to the authors. Requirements of the software: source code is in C++ language and was compiled on i586 Linux platforms; numerical maximization relies on routines of the GNU Scientific Library  that needs to be installed to compile the program.

## Methods

### Markov model

We start off with a simple Markov model for haplotype sequences. This model includes two parameters for each pair of adjacent loci: *a*_*i*_(0,0) = ℙ(*X*_*i*+1 _= 0 | *X*_*i *_= 0) = 1 - *a*_*i*_(0,1) and *a*_*i*_(1, 0) = ℙ(*X*_*i*+1 _= 0 | *X*_*i *_= 1) = 1 - *a*_*i*_(1, 1). We take the maximum likelihood estimate of the parameters given the observed sequences (estimates are smoothed by adding a pseudo-count 0.1 to all counts).

In a Markov model, we found that the subsets of markers with the highest entropy can be determined by a dynamic programming algorithm. Let J*(*k, i*) denote the optimal subset of *k *markers that includes the *i*^th ^marker and *k *- 1 other markers chosen among {1, 2,...,*i *- 1}. The Markov property allows a recursive computation of J*(*k, i*) and its associated entropy using the following algorithm.

• **Initialization**: J*(1, *i*) = {*i*}

• **Recursion**: *H*(*X*_J*(*k, i*)_) = *H*(*X*_J*(*k*-1, *i*')_) + *H*(*X*_*i *_| *X*_*i*'_) where *i*' = arg max_*i*'<*i *_{*H*(*X*_J*(*k*-1, *i*')_) + *H*(*X*_*i *_| *X*_*i*'_)}

• **Backtracking**: the best subset of *m *markers J*(*m*) = argmax_*i *_*H*(*X*_J*(*m, i*)_) is obtained by backtracking.

The time complexity of the algorithm to find J*(*m*) is proportional to *n*^2 ^× *m *and its memory requirement is proportional to *n *× *m*.

### Hidden Markov models

Despite the existence of a relatively fast algorithm that finds optimal tag sets with respect to maximum entropy, the above Markov model suffers from being too simple to sufficiently describe the complicated LD structure. HMMs are interesting alternatives to Markov models. A HMM consists of two layers. In the context of haplotype modelling, the hidden layer models the decay of LD along the genome due to recombination through a Markov chain *S *with transition parameters *a*_*i*_(*u, v*) = ℙ(*S*_*i*+1 _= *v *| *S*_*i *_= *u*), where *u *and *v *take values in a hidden state space S
 MathType@MTEF@5@5@+=feaafiart1ev1aaatCvAUfKttLearuWrP9MDH5MBPbIqV92AaeXatLxBI9gBamrtHrhAL1wy0L2yHvtyaeHbnfgDOvwBHrxAJfwnaebbnrfifHhDYfgasaacH8akY=wiFfYdH8Gipec8Eeeu0xXdbba9frFj0=OqFfea0dXdd9vqai=hGuQ8kuc9pgc9s8qqaq=dirpe0xb9q8qiLsFr0=vr0=vr0dc8meaabaqaciaacaGaaeqabaWaaeGaeaaakeaaimaacqWFse=uaaa@3845@. In the observation layer, the sequence *X *is "emitted" given the underlying haplotype "backbone" *S *according to the emission parameters *b*_*i*_(*x*;*u*) = ℙ(*X*_*i *_= *x *| *S*_*i *_= *u*), *u *∈ S
 MathType@MTEF@5@5@+=feaafiart1ev1aaatCvAUfKttLearuWrP9MDH5MBPbIqV92AaeXatLxBI9gBamrtHrhAL1wy0L2yHvtyaeHbnfgDOvwBHrxAJfwnaebbnrfifHhDYfgasaacH8akY=wiFfYdH8Gipec8Eeeu0xXdbba9frFj0=OqFfea0dXdd9vqai=hGuQ8kuc9pgc9s8qqaq=dirpe0xb9q8qiLsFr0=vr0=vr0dc8meaabaqaciaacaGaaeqabaWaaeGaeaaakeaaimaacqWFse=uaaa@3845@, *x *∈ {0,1}. The maximum likelihood estimates of the parameters can be obtained by the classical Expectation-Maximization (EM) algorithm [42-44].

Some HMMs for haplotype sequences have already been introduced for purposes other than marker selection. We will compare results obtained with two HMMs: the model of Daly *et al*. [10] and the models of Li and Stephens [22]. For the sake of completeness, we also include a simple HMM embedding the Markov model described in the previous section.

#### Efficient bottom-up selection of tag SNPs in HMM

We adopt a simple bottom-up strategy to select subsets of markers. Of course, this cannot ensure an optimal solution. Starting with an empty set of markers, we add markers one by one in such a way that the gain of information content is maximized at each step. Let *J *denote the current set of markers. We want to add the marker i* that maximizes *H*(*X*_*J*∪{*i*}_). This marker i* also maximizes *H*(*X*_*i *_| *X*_*J*_) since *H*(*X*_*J*∪{*i*}_) = *H*(*X*_*i *_| *X*_*J*_) + *H*(*X*_*J*_).

In the HMM framework, exact computation of the conditional entropy *H*(*X*_*i *_| *X*_*J*_) is not tractable but we will now describe an efficient way to approximate it. Using a sample (*x*^(1)^,..., *x*^(*K*)^) of *K *sequences simulated from the HMM, we can approximate

*H *(*X*_*i *_| *X*_*J*_) = EXJ
 MathType@MTEF@5@5@+=feaafiart1ev1aaatCvAUfKttLearuWrP9MDH5MBPbIqV92AaeXatLxBI9gBaebbnrfifHhDYfgasaacH8akY=wiFfYdH8Gipec8Eeeu0xXdbba9frFj0=OqFfea0dXdd9vqai=hGuQ8kuc9pgc9s8qqaq=dirpe0xb9q8qiLsFr0=vr0=vr0dc8meaabaqaciaacaGaaeqabaqabeGadaaakeaatuuDJXwAK1uy0HMmaeHbfv3ySLgzG0uy0HgiuD3BaGabaiab=ri8fnaaBaaaleaaieGacqGFybawdaWgaaadbaGae4NsaOeabeaaaSqabaaaaa@3B47@(∑_*y*∈{0,1} _ℙ(*X*_*i *_= *y *| *X*_*J*_) log ℙ(*X*_*i *_= *y *| *X*_*J*_)) by

H(Xi|Xj)≈1K∑k=1K∑y∈{0,1}ℙ(Xi=y|XJ=xJ(k))log⁡ℙ(Xi=y|XJ=xJ(k)).
 MathType@MTEF@5@5@+=feaafiart1ev1aaatCvAUfKttLearuWrP9MDH5MBPbIqV92AaeXatLxBI9gBaebbnrfifHhDYfgasaacH8akY=wiFfYdH8Gipec8Eeeu0xXdbba9frFj0=OqFfea0dXdd9vqai=hGuQ8kuc9pgc9s8qqaq=dirpe0xb9q8qiLsFr0=vr0=vr0dc8meaabaqaciaacaGaaeqabaqabeGadaaakeaacqWGibascqGGOaakcqWGybawdaWgaaWcbaGaemyAaKgabeaakiabcYha8jabdIfaynaaBaaaleaacqWGQbGAaeqaaOGaeiykaKIaeyisIS7aaSaaaeaacqaIXaqmaeaacqWGlbWsaaWaaabCaeaadaaeqbqaamrr1ngBPrwtHrhAYaqeguuDJXwAKbstHrhAGq1DVbaceaGae8xgHaLaeiikaGIaemiwaG1aaSbaaSqaaiabdMgaPbqabaGccqGH9aqpcqWG5bqEcqGG8baFcqWGybawdaWgaaWcbaGaemOsaOeabeaakiabg2da9iabdIha4naaDaaaleaacqWGkbGsaeaacqGGOaakcqWGRbWAcqGGPaqkaaGccqGGPaqkcyGGSbaBcqGGVbWBcqGGNbWzcqWFzecucqGGOaakcqWGybawdaWgaaWcbaGaemyAaKgabeaakiabg2da9iabdMha5jabcYha8jabdIfaynaaBaaaleaacqWGkbGsaeqaaOGaeyypa0JaemiEaG3aa0baaSqaaiabdQeakbqaaiabcIcaOiabdUgaRjabcMcaPaaakiabcMcaPaWcbaGaemyEaKNaeyicI4Saei4EaSNaeGimaaJaeiilaWIaeGymaeJaeiyFa0habeqdcqGHris5aaWcbaGaem4AaSMaeyypa0JaeGymaedabaGaem4saSeaniabggHiLdGccqGGUaGlaaa@8099@

The central limit theorem states that the standard deviation of the error decreases asymptotically at a rate of order 1/K
 MathType@MTEF@5@5@+=feaafiart1ev1aaatCvAUfKttLearuWrP9MDH5MBPbIqV92AaeXatLxBI9gBaebbnrfifHhDYfgasaacH8akY=wiFfYdH8Gipec8Eeeu0xXdbba9frFj0=OqFfea0dXdd9vqai=hGuQ8kuc9pgc9s8qqaq=dirpe0xb9q8qiLsFr0=vr0=vr0dc8meaabaqaciaacaGaaeqabaqabeGadaaakeaadaGcaaqaaiabdUealbWcbeaaaaa@2DE6@. The term ℙ(*X*_*i *_= *y *| *X*_*J *_= xJ(k)
 MathType@MTEF@5@5@+=feaafiart1ev1aaatCvAUfKttLearuWrP9MDH5MBPbIqV92AaeXatLxBI9gBaebbnrfifHhDYfgasaacH8akY=wiFfYdH8Gipec8Eeeu0xXdbba9frFj0=OqFfea0dXdd9vqai=hGuQ8kuc9pgc9s8qqaq=dirpe0xb9q8qiLsFr0=vr0=vr0dc8meaabaqaciaacaGaaeqabaqabeGadaaakeaacqWG4baEdaqhaaWcbaGaemOsaOeabaGaeiikaGIaem4AaSMaeiykaKcaaaaa@3280@) is equal to ∑_*u *_*b*(*y*;*u*)ℙ(*S*_*i *_= *u *| *X*_*J *_= xJ(k)
 MathType@MTEF@5@5@+=feaafiart1ev1aaatCvAUfKttLearuWrP9MDH5MBPbIqV92AaeXatLxBI9gBaebbnrfifHhDYfgasaacH8akY=wiFfYdH8Gipec8Eeeu0xXdbba9frFj0=OqFfea0dXdd9vqai=hGuQ8kuc9pgc9s8qqaq=dirpe0xb9q8qiLsFr0=vr0=vr0dc8meaabaqaciaacaGaaeqabaqabeGadaaakeaacqWG4baEdaqhaaWcbaGaemOsaOeabaGaeiikaGIaem4AaSMaeiykaKcaaaaa@3280@), where ℙ(*S*_*i*_*= u *| *X*_*J *_= xJ(k)
 MathType@MTEF@5@5@+=feaafiart1ev1aaatCvAUfKttLearuWrP9MDH5MBPbIqV92AaeXatLxBI9gBaebbnrfifHhDYfgasaacH8akY=wiFfYdH8Gipec8Eeeu0xXdbba9frFj0=OqFfea0dXdd9vqai=hGuQ8kuc9pgc9s8qqaq=dirpe0xb9q8qiLsFr0=vr0=vr0dc8meaabaqaciaacaGaaeqabaqabeGadaaakeaacqWG4baEdaqhaaWcbaGaemOsaOeabaGaeiikaGIaem4AaSMaeiykaKcaaaaa@3280@) can be obtained for all *i *by a single pass of the forward-backward algorithm [44] (time complexity proportional to *n*). In principle, ℙ(*S*_*i *_= *u *| *X*_*J *_= *X*_*J*_xJ(k)
 MathType@MTEF@5@5@+=feaafiart1ev1aaatCvAUfKttLearuWrP9MDH5MBPbIqV92AaeXatLxBI9gBaebbnrfifHhDYfgasaacH8akY=wiFfYdH8Gipec8Eeeu0xXdbba9frFj0=OqFfea0dXdd9vqai=hGuQ8kuc9pgc9s8qqaq=dirpe0xb9q8qiLsFr0=vr0=vr0dc8meaabaqaciaacaGaaeqabaqabeGadaaakeaacqWG4baEdaqhaaWcbaGaemOsaOeabaGaeiikaGIaem4AaSMaeiykaKcaaaaa@3280@) should be recomputed for all *i *after each addition of a SNP to *J*. The whole bottom-up selection algorithm would then be of complexity *n*^2^. However, it is clear that adding a SNP i* somewhere does not change the informativeness of SNPs sufficiently far away. Consequently, it is unnecessary to update ℙ(*S*_*i *_= *u *| *X*_*J *_= xJ(k)
 MathType@MTEF@5@5@+=feaafiart1ev1aaatCvAUfKttLearuWrP9MDH5MBPbIqV92AaeXatLxBI9gBaebbnrfifHhDYfgasaacH8akY=wiFfYdH8Gipec8Eeeu0xXdbba9frFj0=OqFfea0dXdd9vqai=hGuQ8kuc9pgc9s8qqaq=dirpe0xb9q8qiLsFr0=vr0=vr0dc8meaabaqaciaacaGaaeqabaqabeGadaaakeaacqWG4baEdaqhaaWcbaGaemOsaOeabaGaeiikaGIaem4AaSMaeiykaKcaaaaa@3280@) for all *i*. The distance from which the terms do not need to be recomputed depends on a complex balance between factors such as the level of LD around i*, the set *J*, the particular haplotype *x*^(*k*) ^and the level of approximation we accept. It is remarkable that the terms ℙ(*S*_*i *_= *u *| *X*_*J *_= xJ(k)
 MathType@MTEF@5@5@+=feaafiart1ev1aaatCvAUfKttLearuWrP9MDH5MBPbIqV92AaeXatLxBI9gBaebbnrfifHhDYfgasaacH8akY=wiFfYdH8Gipec8Eeeu0xXdbba9frFj0=OqFfea0dXdd9vqai=hGuQ8kuc9pgc9s8qqaq=dirpe0xb9q8qiLsFr0=vr0=vr0dc8meaabaqaciaacaGaaeqabaqabeGadaaakeaacqWG4baEdaqhaaWcbaGaemOsaOeabaGaeiikaGIaem4AaSMaeiykaKcaaaaa@3280@) can be computed in the order they appear on each side of i* (this implies to keep forward and backward terms from the previous iteration of the bottom-up algorithm). Thereby, at each step of the algorithm, we can automatically restrict the computation to the relevant portion of each sequence *x*^(*k*)^. This decreases considerably the time complexity of the bottom-up algorithm for large sequences. In our applications, we take *K *= 500 and we update ℙ(*S*_*i *_= *u *| *X*_*J *_= xJ(k)
 MathType@MTEF@5@5@+=feaafiart1ev1aaatCvAUfKttLearuWrP9MDH5MBPbIqV92AaeXatLxBI9gBaebbnrfifHhDYfgasaacH8akY=wiFfYdH8Gipec8Eeeu0xXdbba9frFj0=OqFfea0dXdd9vqai=hGuQ8kuc9pgc9s8qqaq=dirpe0xb9q8qiLsFr0=vr0=vr0dc8meaabaqaciaacaGaaeqabaqabeGadaaakeaacqWG4baEdaqhaaWcbaGaemOsaOeabaGaeiikaGIaem4AaSMaeiykaKcaaaaa@3280@) only for those *i *that are close enough from i* such that max_*u *_|ℙ(*S*_*i *_= *u*|*X*_*j*' _=xJ′(q)
 MathType@MTEF@5@5@+=feaafiart1ev1aaatCvAUfKttLearuWrP9MDH5MBPbIqV92AaeXatLxBI9gBaebbnrfifHhDYfgasaacH8akY=wiFfYdH8Gipec8Eeeu0xXdbba9frFj0=OqFfea0dXdd9vqai=hGuQ8kuc9pgc9s8qqaq=dirpe0xb9q8qiLsFr0=vr0=vr0dc8meaabaqaciaacaGaaeqabaqabeGadaaakeaacqWG4baEdaqhaaWcbaGafmOsaOKbauaaaeaacqGGOaakcqWGXbqCcqGGPaqkaaaaaa@3298@) - ℙ(*S*_*i *_= *u*|*X*_*J *_= xJ(q)
 MathType@MTEF@5@5@+=feaafiart1ev1aaatCvAUfKttLearuWrP9MDH5MBPbIqV92AaeXatLxBI9gBaebbnrfifHhDYfgasaacH8akY=wiFfYdH8Gipec8Eeeu0xXdbba9frFj0=OqFfea0dXdd9vqai=hGuQ8kuc9pgc9s8qqaq=dirpe0xb9q8qiLsFr0=vr0=vr0dc8meaabaqaciaacaGaaeqabaqabeGadaaakeaacqWG4baEdaqhaaWcbaGaemOsaOeabaGaeiikaGIaemyCaeNaeiykaKcaaaaa@328C@)| > 0.001, where *J*' = {*J *∪ i*}.

#### From unconstrained HMMs to Li and Stephens' models

The first HMM we consider is a two-state heterogeneous HMM, which is the most general with two hidden states |S
 MathType@MTEF@5@5@+=feaafiart1ev1aaatCvAUfKttLearuWrP9MDH5MBPbIqV92AaeXatLxBI9gBamrtHrhAL1wy0L2yHvtyaeHbnfgDOvwBHrxAJfwnaebbnrfifHhDYfgasaacH8akY=wiFfYdH8Gipec8Eeeu0xXdbba9frFj0=OqFfea0dXdd9vqai=hGuQ8kuc9pgc9s8qqaq=dirpe0xb9q8qiLsFr0=vr0=vr0dc8meaabaqaciaacaGaaeqabaWaaeGaeaaakeaaimaacqWFse=uaaa@3845@| = 2. For each SNP site, there are |S
 MathType@MTEF@5@5@+=feaafiart1ev1aaatCvAUfKttLearuWrP9MDH5MBPbIqV92AaeXatLxBI9gBamrtHrhAL1wy0L2yHvtyaeHbnfgDOvwBHrxAJfwnaebbnrfifHhDYfgasaacH8akY=wiFfYdH8Gipec8Eeeu0xXdbba9frFj0=OqFfea0dXdd9vqai=hGuQ8kuc9pgc9s8qqaq=dirpe0xb9q8qiLsFr0=vr0=vr0dc8meaabaqaciaacaGaaeqabaWaaeGaeaaakeaaimaacqWFse=uaaa@3845@| × (|S
 MathType@MTEF@5@5@+=feaafiart1ev1aaatCvAUfKttLearuWrP9MDH5MBPbIqV92AaeXatLxBI9gBamrtHrhAL1wy0L2yHvtyaeHbnfgDOvwBHrxAJfwnaebbnrfifHhDYfgasaacH8akY=wiFfYdH8Gipec8Eeeu0xXdbba9frFj0=OqFfea0dXdd9vqai=hGuQ8kuc9pgc9s8qqaq=dirpe0xb9q8qiLsFr0=vr0=vr0dc8meaabaqaciaacaGaaeqabaWaaeGaeaaakeaaimaacqWFse=uaaa@3845@| - 1) transition parameters and |S
 MathType@MTEF@5@5@+=feaafiart1ev1aaatCvAUfKttLearuWrP9MDH5MBPbIqV92AaeXatLxBI9gBamrtHrhAL1wy0L2yHvtyaeHbnfgDOvwBHrxAJfwnaebbnrfifHhDYfgasaacH8akY=wiFfYdH8Gipec8Eeeu0xXdbba9frFj0=OqFfea0dXdd9vqai=hGuQ8kuc9pgc9s8qqaq=dirpe0xb9q8qiLsFr0=vr0=vr0dc8meaabaqaciaacaGaaeqabaWaaeGaeaaakeaaimaacqWFse=uaaa@3845@| emission parameters in an unconstrained HMM. With a practical sample size, we can only deal with unconstrained HMMs of a few hidden states. In order to use HMMs with large number of hidden states, we need to reduce the number of free parameters by imposing constraints on transition and emission probabilities. Daly *et al*. [10] introduced a constrained HMM with four hidden states |S
 MathType@MTEF@5@5@+=feaafiart1ev1aaatCvAUfKttLearuWrP9MDH5MBPbIqV92AaeXatLxBI9gBamrtHrhAL1wy0L2yHvtyaeHbnfgDOvwBHrxAJfwnaebbnrfifHhDYfgasaacH8akY=wiFfYdH8Gipec8Eeeu0xXdbba9frFj0=OqFfea0dXdd9vqai=hGuQ8kuc9pgc9s8qqaq=dirpe0xb9q8qiLsFr0=vr0=vr0dc8meaabaqaciaacaGaaeqabaWaaeGaeaaakeaaimaacqWFse=uaaa@3845@| = 4 to model SNP sequences. As unconstrained models, this model has |S
 MathType@MTEF@5@5@+=feaafiart1ev1aaatCvAUfKttLearuWrP9MDH5MBPbIqV92AaeXatLxBI9gBamrtHrhAL1wy0L2yHvtyaeHbnfgDOvwBHrxAJfwnaebbnrfifHhDYfgasaacH8akY=wiFfYdH8Gipec8Eeeu0xXdbba9frFj0=OqFfea0dXdd9vqai=hGuQ8kuc9pgc9s8qqaq=dirpe0xb9q8qiLsFr0=vr0=vr0dc8meaabaqaciaacaGaaeqabaWaaeGaeaaakeaaimaacqWFse=uaaa@3845@| emission parameters per SNP site but transition probabilities are highly constrained: instead of |S
 MathType@MTEF@5@5@+=feaafiart1ev1aaatCvAUfKttLearuWrP9MDH5MBPbIqV92AaeXatLxBI9gBamrtHrhAL1wy0L2yHvtyaeHbnfgDOvwBHrxAJfwnaebbnrfifHhDYfgasaacH8akY=wiFfYdH8Gipec8Eeeu0xXdbba9frFj0=OqFfea0dXdd9vqai=hGuQ8kuc9pgc9s8qqaq=dirpe0xb9q8qiLsFr0=vr0=vr0dc8meaabaqaciaacaGaaeqabaWaaeGaeaaakeaaimaacqWFse=uaaa@3845@| × (|S
 MathType@MTEF@5@5@+=feaafiart1ev1aaatCvAUfKttLearuWrP9MDH5MBPbIqV92AaeXatLxBI9gBamrtHrhAL1wy0L2yHvtyaeHbnfgDOvwBHrxAJfwnaebbnrfifHhDYfgasaacH8akY=wiFfYdH8Gipec8Eeeu0xXdbba9frFj0=OqFfea0dXdd9vqai=hGuQ8kuc9pgc9s8qqaq=dirpe0xb9q8qiLsFr0=vr0=vr0dc8meaabaqaciaacaGaaeqabaWaaeGaeaaakeaaimaacqWFse=uaaa@3845@| - 1) transition parameters per SNP site, a single parameter is used. Namely, all the transitions associated with a change of backbone are modeled as having the same probability. This model includes more haplotype backbones and is more meaningful than the unconstrained two-state model. However, the choice of the number of hidden states is mainly arbitrary, even though four may be adequate to the particular region studied by Daly *et al*. [10]. Besides, at each position the four haplotype backbones are modeled as having the same marginal probability. Li and Stephens [22] introduced an attractive generalization of Daly's model that bypasses the choice of the number of hidden states. Given *Q *previously observed sequences *x *= (*x*^(1)^, *x*^(2)^,..., *x*^(*Q*)^), their HMM models an additional sequence as a mosaic of segments sampled from these *Q *sequences. In this model, the hidden variable *S*_*i *_corresponds to the template sequence at locus *i*. Although the number of hidden states is as large as *Q*, a single parameter *α *is introduced to account for the recombination intensity:

ai(u,v)={exp⁡(−αdi/Q)+(1−exp⁡(−αdi/Q))/Qif u=v(1−exp⁡(1−αdi/Q))/Qotherwise,
 MathType@MTEF@5@5@+=feaafiart1ev1aaatCvAUfKttLearuWrP9MDH5MBPbIqV92AaeXatLxBI9gBaebbnrfifHhDYfgasaacH8akY=wiFfYdH8Gipec8Eeeu0xXdbba9frFj0=OqFfea0dXdd9vqai=hGuQ8kuc9pgc9s8qqaq=dirpe0xb9q8qiLsFr0=vr0=vr0dc8meaabaqaciaacaGaaeqabaqabeGadaaakeaacqWGHbqydaWgaaWcbaGaemyAaKgabeaakiabcIcaOiabdwha1jabcYcaSiabdAha2jabcMcaPiabg2da9maaceaabaqbaeaabiGaaaqaaiGbcwgaLjabcIha4jabcchaWjabcIcaOiabgkHiTGGaciab=f7aHjabdsgaKnaaBaaaleaacqWGPbqAaeqaaOGaei4la8IaemyuaeLaeiykaKIaey4kaSIaeiikaGIaeGymaeJaeyOeI0IagiyzauMaeiiEaGNaeiiCaaNaeiikaGIaeyOeI0Iae8xSdeMaemizaq2aaSbaaSqaaiabdMgaPbqabaGccqGGVaWlcqWGrbqucqGGPaqkcqGGPaqkcqGGVaWlcqWGrbquaeaacqqGPbqAcqqGMbGzcqqGGaaicqWG1bqDcqGH9aqpcqWG2bGDaeaacqGGOaakcqaIXaqmcqGHsislcyGGLbqzcqGG4baEcqGGWbaCcqGGOaakcqaIXaqmcqGHsislcqWFXoqycqWGKbazdaWgaaWcbaGaemyAaKgabeaakiabc+caViabdgfarjabcMcaPiabcMcaPiabc+caViabdgfarbqaaiabb+gaVjabbsha0jabbIgaOjabbwgaLjabbkhaYjabbEha3jabbMgaPjabbohaZjabbwgaLjabcYcaSaaaaiaawUhaaaaa@8123@

where *d*_*i *_denotes the physical distance between loci *i *and *i *+ 1. Emission probabilities model possible differences (point mutations) at each position *i *between the new sequence and its local template sequence *u *using a single parameter *β *= ℙ(*X*_*i *_≠ xi(u)
 MathType@MTEF@5@5@+=feaafiart1ev1aaatCvAUfKttLearuWrP9MDH5MBPbIqV92AaeXatLxBI9gBaebbnrfifHhDYfgasaacH8akY=wiFfYdH8Gipec8Eeeu0xXdbba9frFj0=OqFfea0dXdd9vqai=hGuQ8kuc9pgc9s8qqaq=dirpe0xb9q8qiLsFr0=vr0=vr0dc8meaabaqaciaacaGaaeqabaqabeGadaaakeaacqWG4baEdaqhaaWcbaGaemyAaKgabaGaeiikaGIaemyDauNaeiykaKcaaaaa@32D2@ |*S*_*i *_= *u*) (a slightly different parametrization is found in the original paper).

A version of this HMM accounting for recombination hot-spots and other recombination rates heterogeneities has also been proposed [22]. Instead of a constant *α*, different *α*_*i*_'s are allowed along the genome. Following Li and Stephens, we impose a prior distribution for the *α*_*i*_'s by setting *a*_*i *_= *γλ*_*i*_, where *γ *does not depend on *i *and log_10_(*λ*_*i*_)~N
 MathType@MTEF@5@5@+=feaafiart1ev1aaatCvAUfKttLearuWrP9MDH5MBPbIqV92AaeXatLxBI9gBamrtHrhAL1wy0L2yHvtyaeHbnfgDOvwBHrxAJfwnaebbnrfifHhDYfgasaacH8akY=wiFfYdH8Gipec8Eeeu0xXdbba9frFj0=OqFfea0dXdd9vqai=hGuQ8kuc9pgc9s8qqaq=dirpe0xb9q8qiLsFr0=vr0=vr0dc8meaabaqaciaacaGaaeqabaWaaeGaeaaakeaaimaacqWFneVtaaa@383B@(0,0.5^2^). This choice puts the *α*_*i*_'s values in the neighborhood of a baseline level *γ*, allowing the recombination intensity to vary up and down by one order of magnitude. Li and Stephens [22] were mainly interested in estimating *α *or the *α*_*i*_'s and in how the estimates relate to the recombination parameter *ρ *of the coalescent model. This motivation lead them to propose two computationally intensive estimation procedures referred as PAC-A and PAC-B in their paper. Here the aim is to fit a model for the occurrence of an additional sequence and thus it makes more sense to maximize ∏q=1Qℙθ,x(−q)(x(q))
MathType@MTEF@5@5@+=feaafiart1ev1aaatCvAUfKttLearuWrP9MDH5MBPbIqV92AaeXatLxBI9gBaebbnrfifHhDYfgasaacH8akY=wiFfYdH8Gipec8Eeeu0xXdbba9frFj0=OqFfea0dXdd9vqai=hGuQ8kuc9pgc9s8qqaq=dirpe0xb9q8qiLsFr0=vr0=vr0dc8meaabaqaciaacaGaaeqabaqabeGadaaakeaadaqeWaqaamrr1ngBPrwtHrhAYaqeguuDJXwAKbstHrhAGq1DVbaceaGae8xgHa1aaSbaaSqaaGGaciab+H7aXjabcYcaSiabdIha4naaCaaameqabaGaeiikaGIaeyOeI0IaemyCaeNaeiykaKcaaaWcbeaaaeaacqWGXbqCcqGH9aqpcqaIXaqmaeaacqWGrbqua0Gaey4dIunakiabcIcaOiabdIha4naaCaaaleqabaGaeiikaGIaemyCaeNaeiykaKcaaOGaeiykaKcaaa@4CDC@, where *θ *= (*α,β*) and *x*^(-*q*) ^denotes all the *Q *observed sequences but *q*. This maximization is related to the pseudo-likelihood methods introduced by Besag [45]. We developed an EM-like algorithm to obtain the value of *θ *that maximizes this quantity. The *k*^*th *^iteration of our algorithm consists in getting *θ*^(*k*) ^= (*α*^(*k*)^, *β*^(*k*)^) that maximizes Eθ(k−1)(log⁡∏q=1Qℙθ,x(−q)(x(q),S)|x)
MathType@MTEF@5@5@+=feaafiart1ev1aaatCvAUfKttLearuWrP9MDH5MBPbIqV92AaeXatLxBI9gBaebbnrfifHhDYfgasaacH8akY=wiFfYdH8Gipec8Eeeu0xXdbba9frFj0=OqFfea0dXdd9vqai=hGuQ8kuc9pgc9s8qqaq=dirpe0xb9q8qiLsFr0=vr0=vr0dc8meaabaqaciaacaGaaeqabaqabeGadaaakeaacqWGfbqrdaWgaaWcbaacciGae8hUde3aaWbaaWqabeaacqGGOaakcqWGRbWAcqGHsislcqaIXaqmcqGGPaqkaaaaleqaaOGaeiikaGIagiiBaWMaei4Ba8Maei4zaC2aaebmaeaatuuDJXwAK1uy0HMmaeHbfv3ySLgzG0uy0HgiuD3BaGabaiab+LriqnaaBaaaleaacqWF4oqCcqGGSaalcqWG4baEdaahaaadbeqaaiabcIcaOiabgkHiTiabdghaXjabcMcaPaaaaSqabaaabaGaemyCaeNaeyypa0JaeGymaedabaGaemyuaefaniabg+GivdGccqGGOaakcqWG4baEdaahaaWcbeqaaiabcIcaOiabdghaXjabcMcaPaaakiabcYcaSiabdofatjabcMcaPiabcYha8jabdIha4jabcMcaPaaa@5FD5@ as

α(k)=arg⁡max⁡α[∑i=1n−1log⁡((1−exp⁡(−αdi/Q′))/Q′)∑q=1Q∑(u,v),u≠vℙθ(k−1),x(−q)(Si=u,Si+1=v|x(q))     +∑i=1n−1log⁡(exp⁡(−αdi/Q′)+(1−exp⁡(−αdi/Q′))/Q′)∑q=1Q∑uℙθ(k−1),x(−q)(Si=u,Si+1=v|x(q))]β(k)=1Q1n∑i=1n∑q=1Q∑u≠qI{xi≠xu}ℙθ(k−1),x(−q)(Si=u|x(q)),
 MathType@MTEF@5@5@+=feaafiart1ev1aaatCvAUfKttLearuWrP9MDH5MBPbIqV92AaeXatLxBI9gBaebbnrfifHhDYfgasaacH8akY=wiFfYdH8Gipec8Eeeu0xXdbba9frFj0=OqFfea0dXdd9vqai=hGuQ8kuc9pgc9s8qqaq=dirpe0xb9q8qiLsFr0=vr0=vr0dc8meaabaqaciaacaGaaeqabaqabeGadaaakeGabaaGeuaabmqaceaaaqGabeGaeaibeaSbbaacciGae8xSde2aaWbaaSqabeaacqGGOaakcqWGRbWAcqGGPaqkaaGccqGH9aqpcyGGHbqycqGGYbGCcqGGNbWzdaWfqaqaaiGbc2gaTjabcggaHjabcIha4bWcbaGae8xSdegabeaakiabcUfaBnaaqahabaGagiiBaWMaei4Ba8Maei4zaCMaeiikaGIaeiikaGIaeGymaeJaeyOeI0IagiyzauMaeiiEaGNaeiiCaaNaeiikaGIaeyOeI0Iae8xSdeMaemizaq2aaSbaaSqaaiabdMgaPbqabaaabaGaemyAaKMaeyypa0JaeGymaedabaGaemOBa4MaeyOeI0IaeGymaedaniabggHiLdGccqGGVaWlcuWGrbqugaqbaiabcMcaPiabcMcaPiabc+caViqbdgfarzaafaGaeiykaKYaaabCaeaadaaeqbqaamrr1ngBPrwtHrhAYaqeguuDJXwAKbstHrhAGq1DVbaceaGae4xgHa1aaSbaaSqaaiab=H7aXnaaCaaameqabaGaeiikaGIaem4AaSMaeyOeI0IaeGymaeJaeiykaKcaaSGaeiilaWIaemiEaG3aaWbaaWqabeaacqGGOaakcqGHsislcqWGXbqCcqGGPaqkaaaaleqaaaqaaiabcIcaOiabdwha1jabcYcaSiabdAha2jabcMcaPiabcYcaSiabdwha1jabgcMi5kabdAha2bqab0GaeyyeIuoaaSqaaiabdghaXjabg2da9iabigdaXaqaaiabdgfarbqdcqGHris5aOGaeiikaGIaem4uam1aaSbaaSqaaiabdMgaPbqabaGccqGH9aqpcqWG1bqDcqGGSaalcqWGtbWudaWgaaWcbaGaemyAaKMaey4kaSIaeGymaedabeaakiabg2da9iabdAha2jabcYha8jabdIha4naaCaaaleqabaGaeiikaGIaemyCaeNaeiykaKcaaOGaeiykaKcabaGaaCzcaiaaxMaacqGHRaWkdaaeWbqaaiGbcYgaSjabc+gaVjabcEgaNjabcIcaOiGbcwgaLjabcIha4jabcchaWjabcIcaOiabgkHiTiab=f7aHjabdsgaKnaaBaaaleaacqWGPbqAaeqaaaqaaiabdMgaPjabg2da9iabigdaXaqaaiabd6gaUjabgkHiTiabigdaXaqdcqGHris5aOGaei4la8IafmyuaeLbauaacqGGPaqkcqGHRaWkcqGGOaakcqaIXaqmcqGHsislcyGGLbqzcqGG4baEcqGGWbaCcqGGOaakcqGHsislcqWFXoqycqWGKbazdaWgaaWcbaGaemyAaKgabeaakiabc+caViqbdgfarzaafaGaeiykaKIaeiykaKIaei4la8IafmyuaeLbauaacqGGPaqkdaaeWbqaamaaqafabaGae4xgHa1aaSbaaSqaaiab=H7aXnaaCaaameqabaGaeiikaGIaem4AaSMaeyOeI0IaeGymaeJaeiykaKcaaSGaeiilaWIaemiEaG3aaWbaaWqabeaacqGGOaakcqGHsislcqWGXbqCcqGGPaqkaaaaleqaaaqaaiabdwha1bqab0GaeyyeIuoaaSqaaiabdghaXjabg2da9iabigdaXaqaaiabdgfarbqdcqGHris5aOGaeiikaGIaem4uam1aaSbaaSqaaiabdMgaPbqabaGccqGH9aqpcqWG1bqDcqGGSaalcqWGtbWudaWgaaWcbaGaemyAaKMaey4kaSIaeGymaedabeaakiabg2da9iabdAha2jabcYha8jabdIha4naaCaaaleqabaGaeiikaGIaemyCaeNaeiykaKcaaOGaeiykaKIaeiyxa0faaeaacqWFYoGydaahaaWcbeqaaiabcIcaOiabdUgaRjabcMcaPaaakiabg2da9maalaaabaGaeGymaedabaGaemyuaefaamaalaaabaGaeGymaedabaGaemOBa4gaamaaqahabaWaaabCaeaadaaeqbqaaiab+Hi8jjabcUha7jabdIha4naaBaaaleaacqWGPbqAaeqaaOGaeyiyIKRaemiEaG3aaSbaaSqaaiabdwha1bqabaGccqGG9bqFcqGFzecudaWgaaWcbaGae8hUde3aaWbaaWqabeaacqGGOaakcqWGRbWAcqGHsislcqaIXaqmcqGGPaqkaaWccqGGSaalcqWG4baEdaahaaadbeqaaiabcIcaOiabgkHiTiabdghaXjabcMcaPaaaaSqabaaabaGaemyDauNaeyiyIKRaemyCaehabeqdcqGHris5aaWcbaGaemyCaeNaeyypa0JaeGymaedabaGaemyuaefaniabggHiLdaaleaacqWGPbqAcqGH9aqpcqaIXaqmaeaacqWGUbGBa0GaeyyeIuoakiabcIcaOiabdofatnaaBaaaleaacqWGPbqAaeqaaOGaeyypa0JaemyDauNaeiiFaWNaemiEaG3aaWbaaSqabeaacqGGOaakcqWGXbqCcqGGPaqkaaGccqGGPaqkcqGGSaalaaaaaa@4DA5@

where *Q*' = *Q *- 1 and I
 MathType@MTEF@5@5@+=feaafiart1ev1aaatCvAUfKttLearuWrP9MDH5MBPbIqV92AaeXatLxBI9gBaebbnrfifHhDYfgasaacH8akY=wiFfYdH8Gipec8Eeeu0xXdbba9frFj0=OqFfea0dXdd9vqai=hGuQ8kuc9pgc9s8qqaq=dirpe0xb9q8qiLsFr0=vr0=vr0dc8meaabaqaciaacaGaaeqabaqabeGadaaakeaatuuDJXwAK1uy0HMmaeHbfv3ySLgzG0uy0HgiuD3BaGabaiab=Hi8jbaa@3894@{*x*_*i *_≠ *x*_*u*_} = 1 when *x*_*i *_≠ *x*_*u *_and I
 MathType@MTEF@5@5@+=feaafiart1ev1aaatCvAUfKttLearuWrP9MDH5MBPbIqV92AaeXatLxBI9gBaebbnrfifHhDYfgasaacH8akY=wiFfYdH8Gipec8Eeeu0xXdbba9frFj0=OqFfea0dXdd9vqai=hGuQ8kuc9pgc9s8qqaq=dirpe0xb9q8qiLsFr0=vr0=vr0dc8meaabaqaciaacaGaaeqabaqabeGadaaakeaatuuDJXwAK1uy0HMmaeHbfv3ySLgzG0uy0HgiuD3BaGabaiab=Hi8jbaa@3894@{*x*_*i *_≠ *x*_*u*_} = 0 elsewhere. The terms ℙθ(k−1),x(−q)
 MathType@MTEF@5@5@+=feaafiart1ev1aaatCvAUfKttLearuWrP9MDH5MBPbIqV92AaeXatLxBI9gBaebbnrfifHhDYfgasaacH8akY=wiFfYdH8Gipec8Eeeu0xXdbba9frFj0=OqFfea0dXdd9vqai=hGuQ8kuc9pgc9s8qqaq=dirpe0xb9q8qiLsFr0=vr0=vr0dc8meaabaqaciaacaGaaeqabaqabeGadaaakeaatuuDJXwAK1uy0HMmaeHbfv3ySLgzG0uy0HgiuD3BaGabaiab=LriqnaaBaaaleaaiiGacqGF4oqCdaahaaadbeqaaiabcIcaOiabdUgaRjabgkHiTiabigdaXiabcMcaPaaaliabcYcaSiabdIha4naaCaaameqabaGaeiikaGIaeyOeI0IaemyCaeNaeiykaKcaaaWcbeaaaaa@4508@ (*S*_*i *_= *u*|*x*^(q) ^and ℙθ(k−1),x(−q)
 MathType@MTEF@5@5@+=feaafiart1ev1aaatCvAUfKttLearuWrP9MDH5MBPbIqV92AaeXatLxBI9gBaebbnrfifHhDYfgasaacH8akY=wiFfYdH8Gipec8Eeeu0xXdbba9frFj0=OqFfea0dXdd9vqai=hGuQ8kuc9pgc9s8qqaq=dirpe0xb9q8qiLsFr0=vr0=vr0dc8meaabaqaciaacaGaaeqabaqabeGadaaakeaatuuDJXwAK1uy0HMmaeHbfv3ySLgzG0uy0HgiuD3BaGabaiab=LriqnaaBaaaleaaiiGacqGF4oqCdaahaaadbeqaaiabcIcaOiabdUgaRjabgkHiTiabigdaXiabcMcaPaaaliabcYcaSiabdIha4naaCaaameqabaGaeiikaGIaeyOeI0IaemyCaeNaeiykaKcaaaWcbeaaaaa@4508@ (*S*_*i *_= *u*, *S*_*i*+1 _= *v *| *x*^(*q*)^) are obtained with the forward-backward algorithm for HMMs and *α*^(*k*) ^is found by numerical maximization. Similarly, in the heterogeneous version of the model, the EM-like algorithm is used to maximize ∏q=1Qℙγ,λ,β(x(q)|x(−q))×∏iπ(λi)
MathType@MTEF@5@5@+=feaafiart1ev1aaatCvAUfKttLearuWrP9MDH5MBPbIqV92AaeXatLxBI9gBaebbnrfifHhDYfgasaacH8akY=wiFfYdH8Gipec8Eeeu0xXdbba9frFj0=OqFfea0dXdd9vqai=hGuQ8kuc9pgc9s8qqaq=dirpe0xb9q8qiLsFr0=vr0=vr0dc8meaabaqaciaacaGaaeqabaqabeGadaaakeaadaqeWaqaamrr1ngBPrwtHrhAYaqeguuDJXwAKbstHrhAGq1DVbaceaGae8xgHa1aaSbaaSqaaGGaciab+n7aNjabcYcaSiab+T7aSjabcYcaSiab+j7aIbqabaGccqGGOaakcqWG4baEdaahaaWcbeqaaiabcIcaOiabdghaXjabcMcaPaaaaeaacqWGXbqCcqGH9aqpcqaIXaqmaeaacqWGrbqua0Gaey4dIunakiabcYha8jabdIha4naaCaaaleqabaGaeiikaGIaeyOeI0IaemyCaeNaeiykaKcaaOGaeiykaKIaey41aq7aaebeaeaacqGFapaCaSqaaiabdMgaPbqab0Gaey4dIunakiabcIcaOiab+T7aSnaaBaaaleaacqWGPbqAaeqaaOGaeiykaKcaaa@5E6A@, where ∏_i_*π*(*λ*_*i*_) is the density of the log-normal prior for the *λ*_*i*_'s. At the *k*^th ^iteration of the algorithm, (*γ*^(*k*)^,λ1(k)
 MathType@MTEF@5@5@+=feaafiart1ev1aaatCvAUfKttLearuWrP9MDH5MBPbIqV92AaeXatLxBI9gBaebbnrfifHhDYfgasaacH8akY=wiFfYdH8Gipec8Eeeu0xXdbba9frFj0=OqFfea0dXdd9vqai=hGuQ8kuc9pgc9s8qqaq=dirpe0xb9q8qiLsFr0=vr0=vr0dc8meaabaqaciaacaGaaeqabaqabeGadaaakeaaiiGacqWF7oaBdaqhaaWcbaGaeGymaedabaGaeiikaGIaem4AaSMaeiykaKcaaaaa@3295@,...,λn(k)
 MathType@MTEF@5@5@+=feaafiart1ev1aaatCvAUfKttLearuWrP9MDH5MBPbIqV92AaeXatLxBI9gBaebbnrfifHhDYfgasaacH8akY=wiFfYdH8Gipec8Eeeu0xXdbba9frFj0=OqFfea0dXdd9vqai=hGuQ8kuc9pgc9s8qqaq=dirpe0xb9q8qiLsFr0=vr0=vr0dc8meaabaqaciaacaGaaeqabaqabeGadaaakeaaiiGacqWF7oaBdaqhaaWcbaGaemOBa4gabaGaeiikaGIaem4AaSMaeiykaKcaaaaa@330A@) are choose to maximize

∑i=1n−1log⁡π(λi)+∑i=1n−1log⁡((1−exp⁡(− γλidi/Q′))/Q′)∑q=1Q∑(u,v),u≠vℙθ(k−1),x(−q)(Si=u,Si+1=v|x(q))+∑i=1n−1log⁡(exp⁡(−γλidi/Q′)+(1−exp⁡(−γλidi/Q′))/Q′)∑q=1Q∑uℙθ(k−1),x(−q)(Si=u,Si+1=v|x(q)),
 MathType@MTEF@5@5@+=feaafiart1ev1aaatCvAUfKttLearuWrP9MDH5MBPbIqV92AaeXatLxBI9gBaebbnrfifHhDYfgasaacH8akY=wiFfYdH8Gipec8Eeeu0xXdbba9frFj0=OqFfea0dXdd9vqai=hGuQ8kuc9pgc9s8qqaq=dirpe0xb9q8qiLsFr0=vr0=vr0dc8meaabaqaciaacaGaaeqabaqabeGadaaakeaafaqabeGabaaabaWaaabCaeaacyGGSbaBcqGGVbWBcqGGNbWziiGacqWFapaCcqGGOaakcqWF7oaBdaWgaaWcbaGaemyAaKgabeaakiabcMcaPiabgUcaRmaaqahabaGagiiBaWMaei4Ba8Maei4zaCMaeiikaGIaeiikaGIaeGymaeJaeyOeI0IagiyzauMaeiiEaGNaeiiCaaNaeiikaGIaeyOeI0IaaGjcVlab=n7aNjab=T7aSnaaBaaaleaacqWGPbqAaeqaaOGaemizaq2aaSbaaSqaaiabdMgaPbqabaGccqGGVaWlcuWGrbqugaqbaiabcMcaPiabcMcaPiabc+caViqbdgfarzaafaGaeiykaKcaleaacqWGPbqAcqGH9aqpcqaIXaqmaeaacqWGUbGBcqGHsislcqaIXaqma0GaeyyeIuoakmaaqahabaWaaabuaeaatuuDJXwAK1uy0HMmaeHbfv3ySLgzG0uy0HgiuD3BaGabaiab+LriqnaaBaaaleaacqWF4oqCdaahaaadbeqaaiabcIcaOiabdUgaRjabgkHiTiabigdaXiabcMcaPaaaliabcYcaSiabdIha4naaCaaameqabaGaeiikaGIaeyOeI0IaemyCaeNaeiykaKcaaaWcbeaaaeaacqGGOaakcqWG1bqDcqGGSaalcqWG2bGDcqGGPaqkcqGGSaalcqWG1bqDcqGHGjsUcqWG2bGDaeqaniabggHiLdaaleaacqWGXbqCcqGH9aqpcqaIXaqmaeaacqWGrbqua0GaeyyeIuoaaSqaaiabdMgaPjabg2da9iabigdaXaqaaiabd6gaUjabgkHiTiabigdaXaqdcqGHris5aOGaeiikaGIaem4uam1aaSbaaSqaaiabdMgaPbqabaGccqGH9aqpcqWG1bqDcqGGSaalcqWGtbWudaWgaaWcbaGaemyAaKMaey4kaSIaeGymaedabeaakiabg2da9iabdAha2jabcYha8jabdIha4naaCaaaleqabaGaeiikaGIaemyCaeNaeiykaKcaaOGaeiykaKcabaGaey4kaSYaaabCaeaacyGGSbaBcqGGVbWBcqGGNbWzcqGGOaakcyGGLbqzcqGG4baEcqGGWbaCcqGGOaakcqGHsislcqWFZoWzcqWF7oaBdaWgaaWcbaGaemyAaKgabeaakiabdsgaKnaaBaaaleaacqWGPbqAaeqaaOGaei4la8IafmyuaeLbauaacqGGPaqkaSqaaiabdMgaPjabg2da9iabigdaXaqaaiabd6gaUjabgkHiTiabigdaXaqdcqGHris5aOGaey4kaSIaeiikaGIaeGymaeJaeyOeI0IagiyzauMaeiiEaGNaeiiCaaNaeiikaGIaeyOeI0Iae83SdCMae83UdW2aaSbaaSqaaiabdMgaPbqabaGccqWGKbazdaWgaaWcbaGaemyAaKgabeaakiabc+caViqbdgfarzaafaGaeiykaKIaeiykaKIaei4la8IafmyuaeLbauaacqGGPaqkdaaeWbqaamaaqafabaGae4xgHa1aaSbaaSqaaiab=H7aXnaaCaaameqabaGaeiikaGIaem4AaSMaeyOeI0IaeGymaeJaeiykaKcaaSGaeiilaWIaemiEaG3aaWbaaWqabeaacqGGOaakcqGHsislcqWGXbqCcqGGPaqkaaaaleqaaaqaaiabdwha1bqab0GaeyyeIuoaaSqaaiabdghaXjabg2da9iabigdaXaqaaiabdgfarbqdcqGHris5aOGaeiikaGIaem4uam1aaSbaaSqaaiabdMgaPbqabaGccqGH9aqpcqWG1bqDcqGGSaalcqWGtbWudaWgaaWcbaGaemyAaKMaey4kaSIaeGymaedabeaakiabg2da9iabdAha2jabcYha8jabdIha4naaCaaaleqabaGaeiikaGIaemyCaeNaeiykaKcaaOGaeiykaKIaeiilaWcaaaaa@0FEF@

and the updating formula for *β *is the same as in the homogeneous case. For our chromosome scale data-set, we allow the baseline intensity *α *or *γ *to change every 100 SNPs (approximately 500 Kbp).

### Tag SNP selection through greedy entropy maximization

As an exploratory effort, we drop the linear structure embedded in Markov models and HMMs while sticking to the principle of maximum entropy. According to the bottom-up selection strategy, when adding one marker i* to a subset *J*, this marker should maximizes *H*(*X*_*i *_| *X*_*J*_). Without a specific model such as a Markov or a hidden Markov model, it is impossible to compute *H*(*X*_*i *_| *X*_*J*_). Instead we replace *H*(*X*_*i *_| *X*_*J*_) by the entropy of *X*_*i *_given the one or two SNPs that are the most informative relative to *i *among the SNPs already selected. Namely, we compute min_*j*∈*J *_*H*(*X*_*i *_| *X*_*j*_) (one SNP) or min⁡(j1,j2)∈J
 MathType@MTEF@5@5@+=feaafiart1ev1aaatCvAUfKttLearuWrP9MDH5MBPbIqV92AaeXatLxBI9gBaebbnrfifHhDYfgasaacH8akY=wiFfYdH8Gipec8Eeeu0xXdbba9frFj0=OqFfea0dXdd9vqai=hGuQ8kuc9pgc9s8qqaq=dirpe0xb9q8qiLsFr0=vr0=vr0dc8meaabaqaciaacaGaaeqabaqabeGadaaakeaacyGGTbqBcqGGPbqAcqGGUbGBdaWgaaWcbaGaeiikaGIaemOAaO2aaSbaaWqaaiabigdaXaqabaWccqGGSaalcqWGQbGAdaWgaaadbaGaeGOmaidabeaaliabcMcaPiabgIGiolabdQeakbqabaaaaa@3B39@*H*(*X*_*i *_| Xj1,j2
 MathType@MTEF@5@5@+=feaafiart1ev1aaatCvAUfKttLearuWrP9MDH5MBPbIqV92AaeXatLxBI9gBaebbnrfifHhDYfgasaacH8akY=wiFfYdH8Gipec8Eeeu0xXdbba9frFj0=OqFfea0dXdd9vqai=hGuQ8kuc9pgc9s8qqaq=dirpe0xb9q8qiLsFr0=vr0=vr0dc8meaabaqaciaacaGaaeqabaqabeGadaaakeaacqWGybawdaWgaaWcbaGaemOAaO2aaSbaaWqaaiabigdaXaqabaWccqGGSaalcqWGQbGAdaWgaaadbaGaeGOmaidabeaaaSqabaaaaa@33FD@) (two SNPs). Estimates of *H*(*X*_*i *_| *X*_*j*_) or *H*(*X*_*i *_| Xj1,j2
 MathType@MTEF@5@5@+=feaafiart1ev1aaatCvAUfKttLearuWrP9MDH5MBPbIqV92AaeXatLxBI9gBaebbnrfifHhDYfgasaacH8akY=wiFfYdH8Gipec8Eeeu0xXdbba9frFj0=OqFfea0dXdd9vqai=hGuQ8kuc9pgc9s8qqaq=dirpe0xb9q8qiLsFr0=vr0=vr0dc8meaabaqaciaacaGaaeqabaqabeGadaaakeaacqWGybawdaWgaaWcbaGaemOAaO2aaSbaaWqaaiabigdaXaqabaWccqGGSaalcqWGQbGAdaWgaaadbaGaeGOmaidabeaaaSqabaaaaa@33FD@) are obtained with the formula *H*(*X*_*i *_| *X*_*j*_) ≈ -∑_*x*,*y*_p^
 MathType@MTEF@5@5@+=feaafiart1ev1aaatCvAUfKttLearuWrP9MDH5MBPbIqV92AaeXatLxBI9gBaebbnrfifHhDYfgasaacH8akY=wiFfYdH8Gipec8Eeeu0xXdbba9frFj0=OqFfea0dXdd9vqai=hGuQ8kuc9pgc9s8qqaq=dirpe0xb9q8qiLsFr0=vr0=vr0dc8meaabaqaciaacaGaaeqabaqabeGadaaakeaacuWGWbaCgaqcaaaa@2E25@(*X*_*i *_= *x*, *X*_*j *_= *y*) logp^
 MathType@MTEF@5@5@+=feaafiart1ev1aaatCvAUfKttLearuWrP9MDH5MBPbIqV92AaeXatLxBI9gBaebbnrfifHhDYfgasaacH8akY=wiFfYdH8Gipec8Eeeu0xXdbba9frFj0=OqFfea0dXdd9vqai=hGuQ8kuc9pgc9s8qqaq=dirpe0xb9q8qiLsFr0=vr0=vr0dc8meaabaqaciaacaGaaeqabaqabeGadaaakeaacuWGWbaCgaqcaaaa@2E25@(*X*_*i *_= *x *| *X*_*j *_= *y*), where p^
 MathType@MTEF@5@5@+=feaafiart1ev1aaatCvAUfKttLearuWrP9MDH5MBPbIqV92AaeXatLxBI9gBaebbnrfifHhDYfgasaacH8akY=wiFfYdH8Gipec8Eeeu0xXdbba9frFj0=OqFfea0dXdd9vqai=hGuQ8kuc9pgc9s8qqaq=dirpe0xb9q8qiLsFr0=vr0=vr0dc8meaabaqaciaacaGaaeqabaqabeGadaaakeaacuWGWbaCgaqcaaaa@2E25@ stands for empirically estimated probabilities. As a result, a model whose density factorizes according to a Directed Acyclic Graph (DAG) is built along the way for those SNPs in the tag set. We smooth the estimates of the conditional probabilities by adding 0.1 to all counts. Later on, we refer to these models as the "greedy" models with context-size 1 and context-size 2.

### Other tag SNP selection methods used for comparison

For comparison, we use two SNP tag procedures described in the literature. Both methods select subsets whose sizes depend simultaneously on the thresholds chosen by the user and on the particular set of sequences.

The first method is the block-based dynamic programming algorithm implemented in the HapBlock software [12,13]. The user needs to choose among three criteria to define blocks and five criteria to select the tag SNPs within the blocks. In keeping with [9] and [12], we use the common haplotype criterion to define potential haplotype blocks. Two parameters *α*_*HB *_and *β*_*HB *_play a role in this definition: any region where common haplotypes represent at least a fraction *α*_*HB *_of the sequences can be considered as a block; a common haplotype is defined as a haplotype sequence accounting for more than a fraction *β*_*HB *_of the observed sequences. In keeping with [4,12,15], we also use the diversity criterion to choose the tag SNPs: within each block, the smallest subset of SNPs that can distinguish more than a proportion *α*_*HB *_of the sequences is selected (note that here we use the same threshold as in the block definition). In parallel, we tested another tag SNP selection criterion based on the empirical entropy of the block: the smallest subset of SNPs that can account for a fraction *α*_*HB *_of the original entropy is selected [13].

The second method comes from a recent work by Carlson *et al*. [8]. The method is based on the *r*^2 ^pairwise measure of LD between two loci defined by *r*^2 ^= (*p*_*AB*_* - p*_*A*_*p*_*B*_)^2^/(*p*_*A*_*p*_*a*_*p*_*B*_*p*_*b*_), where *p*_*A *_= 1 - *p*_*a *_and *p*_*B *_= - *p*_*b *_stand for the alleles frequencies at each locus and *p*_*AB *_denotes the joint allele frequency. A greedy algorithm searches for a tag set such that any SNP not in the subset with a minor allele frequency higher than 0.1 has an empirical *r*^2 ^measure with a tag SNP higher than a chosen threshold.

It has been suggested that any set of SNPs approximately evenly spaced along the sequence is a good tag set [46]. To check this statement we use a simple procedure to build-up sets of increasing size whose SNPs are approximately evenly spaced. We start with a single SNP which is the closest to the middle point of the sequence. One SNP at a time, we increase the size of the tag set by (1) finding the longest interval that does not contain an already selected SNP, but does contain at least one unselected SNP; (2) adding the SNP that is the closest to the middle point of this interval.

### Data sets and cross-validation scheme

We assess the performances of the tag SNP selection procedures on several data sets that differ by the number and density of markers as well as by the population sampled.

The data come from the genotype trios available from the international HapMap and ENCODE projects (final phase I data release, June 2005). A genotype trio is made of the genotype of one child and those of its two parents. Trio data make it possible to infer haplotypes by simple Mendelian genetic rules at most genotyped positions (assuming no recombination in the last generation). We use the hap2 program [47] to infer haplotypes at unsolved positions and missing data. This program combines trio information with population information obtained from the entire sample. The phased data sets are available for download [see Additional file 2].

Our genome-wide data consists of two sets of 120 haplotypes: one is sampled from a Yoruba population in Nigeria (YRI data sets), the other comes from a Utah population of European ancestry (CEU data sets). In each population, 30 trios were genotyped at approximately 1,100,000 SNPs roughly evenly spaced along the genome. The 120 independent haplotypes are obtained after discarding children's haplotypes. The average spacing between adjacent SNPs in this genome-wide data set is around 3 kbp. This density is probably not enough to capture the detailed pattern of polymorphism.

The ENCODE project assesses a much higher density of SNPs in the same individuals for ten 500 kbp regions (the ENCODE regions). These ten regions were chosen to represent a wide variety of LD levels. In the final phase I release, the density is about one SNP per 500 bp in most ENCODE regions.

All our evaluations rely on a six fold cross-validation scheme. The 120 haplotypes are randomly split into six disjoint test sets of 20 haplotypes and six complementary training sets of 100 haplotypes. Parameter estimation and tag SNP selection are based on the training sets while evaluations rely on the test sets.

## Authors' contributions

PN designed and implemented the algorithms, carried out the analyses, and wrote the manuscript. FS participated to the design of the study and helped writing the manuscript. LML proposed to address the SNP selection problem as a data compression task, participated to the design of the study and wrote the manuscript. All authors read and approved the final manuscript.

## Supplementary Material

Additional File 1 **Programs for tag SNP selection and tagged SNP prediction using Li and Stephens models**. This tar-gzipped archive includes the Linux i586 binaries, C++ source code, sample data and instructions on how to compile and to use the programs. Click here for file

Additional File 2 **Data Sets**. This tar-gzipped archive includes the haplotype and genotype data sets used in this study. Click here for file

## Figures and Tables

**Table 1:** Model comparison using code-length, or average negative cross-log-likelihood. Code lengths are given in bits. For Chromosome 7 data sets, SNP loci were sub-sampled in ENCODE regions to maintain an uniform coverage of the chromosome.

	Pop.^(*a*)^	SNPs^(*b*)^	Markov	HMM-2^(*c*)^	HMM-4D^(*d*)^	GR-1^(*e*)^	GR-2^(*f*)^	LS-HOM^(*g*)^	**LS-HET**^(*h*)^
Chr. 7	CEU	42835	–	13334.9	9737.9	10898.7	8273.4	6441.5	**6030.6**
Chr. 7	YRI	42790	–	16783.0	13996.9	15252.2	13543.4	9082.5	**8705.6**
ENr112	CEU	1134	378.6	238.4	152.8	127.2	89.2	53.6	**51.4**
ENr112	YRI	1082	489.9	348.9	238.9	225.0	157.8	80.7	**77.9**
ENr131	CEU	1188	454.0	306.7	161.5	151.7	100.2	66.1	**60.2**
ENr131	YRI	1080	439.7	331.9	244.2	227.4	172.4	101.9	**92.7**
ENr113	CEU	1375	478.7	287.8	160.4	120.6	88.8	57.9	**55.9**
ENr113	YRI	1525	597.9	424.9	286.3	228.3	157.5	84.9	**81.7**
ENm010	CEU	706	261.9	187.2	106.6	106.1	83.1	58.8	**56.7**
ENm010	YRI	741	325.5	250.5	175.4	177.8	152.1	106.6	**101.4**
ENm013	CEU	1001	417.5	279.6	132.2	83.7	57.1	38.2	**37.6**
ENm013	YRI	1111	452.1	336.3	211.0	157.0	108.7	63.2	**62.0**
ENm014	CEU	1110	442.1	290.1	140.9	104.6	71.5	55.3	**50.9**
ENm014	YRI	1224	483.2	338.9	237.0	166.5	117.7	71.7	**68.4**
ENr321	CEU	782	243.1	143.2	90.1	90.4	68.9	48.0	**46.0**
ENr321	YRI	1123	458.9	325.5	232.1	199.1	145.0	77.1	**73.9**
ENr232	CEU	627	189.4	117.2	89.9	98.5	82.3	64.1	**58.4**
ENr232	YRI	833	345.9	268.6	206.2	198.8	161.5	102.2	**93.4**
ENr123	CEU	1183	453.8	294.2	175.2	114.6	76.3	47.5	**46.8**
ENr123	YRI	1055	436.6	291.5	206.1	161.3	118.5	67.7	**66.7**
ENr213	CEU	800	323.9	207.1	87.4	82.3	59.5	41.0	**38.5**
ENr213	YRI	1085	418.5	319.9	219.8	178.7	131.6	75.4	**71.6**

^(*a*)^: Population. ^(*b*)^: Number of actually polymorphic SNPs in the population considered. ^(*c*-*h*)^: HMM-2 for the unconstrained two-state HMM; HMM-4D for the Daly *et al*. HMM; LS-HOM and LS-HET for the homogeneous and the heterogeneous Li and Stephens models; GR-1 and GR-2 for the "greedy" models with context sizes 1 and 2.
